# Cordycepin promotes apoptosis in renal carcinoma cells by activating the MKK7-JNK signaling pathway through inhibition of c-FLIP_L_ expression

**DOI:** 10.1371/journal.pone.0186489

**Published:** 2017-10-18

**Authors:** In-Hu Hwang, Seung Yoon Oh, Hyun-Jin Jang, Eunbi Jo, Jong Cheon Joo, Kyung-Bok Lee, Hwa-Seung Yoo, Mi Young Lee, Soo Jung Park, Ik-Soon Jang

**Affiliations:** 1 Department of Physiology, Korea University College of Medicine, Seoul, Republic of Korea; 2 Neuroscience Research Institute, Korea University College of Medicine, Seoul, Republic of Korea; 3 Department of Sasang Constitutional Medicine, Wonkwang University, Iksan, Republic of Korea; 4 Division of Bioconvergence Analysis, Korea Basic Science Institute, Daejeon, Republic of Korea; 5 East-West Cancer Center, Daejeon University, Daejeon, Korea; 6 KM Convergence Research Division, Korea Institute of Oriental Medicine, Daejeon, Korea; 7 Department of Sasang Constitutional Medicine, Woosuk University, Wanju, Jeonbuk, Republic of Korea; University of South Alabama Mitchell Cancer Institute, UNITED STATES

## Abstract

Cellular FLICE inhibitory protein (c-FLIP) is a key anti-apoptotic regulator that associates with the signaling complex downstream of NF-κB, negatively interfering with apoptotic signaling. The role of c-FLIP downregulation by negative regulation of NF-κB signaling during apoptosis is poorly understood. Here, we demonstrate that NF-κB-mediated c-FLIP_L_ negatively regulates the JNK signaling pathway, and that cordycepin treatment of human renal cancer cells leads to apoptosis induction through c-FLIP_L_ inhibition. TNF-α-induced inflammatory microenvironments stimulated NF-κB signaling and the c-FLIP long form (c-FLIP_L_) in TK-10 cells. Specifically, cordycepin inhibited TNF-α-mediated NF-κB activation, which induced renal cancer cell apoptosis. Cordycepin downregulated GADD45B and c-FLIP_L_, but upregulated MKK7 and phospho-JNK, by preventing nuclear mobilization of NF-κB. Furthermore, siRNA-mediated knockdown of GADD45B in cordycepin-treated TK-10 cells considerably increased MKK7 compared to cordycepin alone. siRNA-mediated knockdown of c-FLIP_L_ prevented TNF-α-induced JNK inactivation, whereas c-FLIP_L_ overexpression inhibited cordycepin-mediated JNK activation. The JNK inhibitor SP600125 strongly inhibited Bax expression. In nude mice, cordycepin significantly decreased tumor volume. Taken together, the results indicate that cordycepin inhibits TNF-α-mediated NF-κB/GADD45B signaling, which activates the MKK7-JNK signaling pathway through inhibition of c-FLIP_L_ expression, thus inducing TK-10 cell apoptosis.

## Introduction

c-FLIP, a master anti-apoptotic mediator that acts via preventing the activation of caspase-8/-10 homologue [1], is involved in TRAIL, Fas, TNF-α, and chemotherapeutic drug resistance in various human cancers [2]. c-FLIP has 13 splice variants, three of which are expressed as proteins: c-FLIP_L_ (55 kDa, long form), c-FLIP_S_ (26 kDa, short form), and c-FLIP_R_ (24-kDa form) [1]. These proteins affect other cellular functions, including increasing cell growth rate and decreasing cell differentiation [2,3]. *Cordyceps militaris* is known to possess remarkable immunostimulating, anti-inflammatory, antimicrobial, and tumoricidal activities, with the primary pharmacological activity varying according to the extract ingredients [4,5,6]. Cordycepin potently suppresses NO production in lipopolysaccharide (LPS)-stimulated RAW 264.7 murine macrophages in an adenosine receptor-independent manner [7] and inhibits LPS-induced inflammation by suppressing NF-κB via Akt and p38 inhibition [8]. Growth arrest and DNA damage-inducible beta (GADD45B) mediates the suppression of Jun N-terminal kinase (JNK) signaling by NF-κB, by targeting mitogen-activated protein kinase kinase 7 (MKK7)/JNK kinase 2 (JNKK2) [9]. NF-κB acts as a tumor promoter in inflammation-associated cancers [10]. The pro-apoptotic JNK is the downstream component of major mitogen-activated protein kinase (MAPK) cascades, including the extracellular signal-regulated kinase 1 and 2 (ERK 1 and 2) and p38 cascades. ERK activation is linked with cell growth and survival [11,12]. JNK and p38 MAPK family members function in a cell-type- and context-specific manner to integrate signals that affect proliferation, differentiation, survival, and migration [13,14]. NF-κB exerts its anti-apoptotic activity partly by downregulating JNK activation [15]. GADD45B, a pivotal survival factor downstream of NF-κB, is involved in the crosstalk between NF-κB and JNK and modulates JNK activation by binding to and inhibiting the JNK kinase, MKK7 [16,17].

In this study, TNF-α-mediated simulation of *in vitro* proinflammatory microenvironment was carried out, which increased activation of the transcription factor NF-κB. We investigated the functional mechanisms underlying the NF-κB-mediated c-FLIP_L_ negative regulation of the JNK signaling pathway. The results indicated that cordycepin prevents constitutive NF-κB signaling, resulting in the stimulation of the MKK7-JNK signaling pathway through inhibition of c-FLIP_L_ expression and the consequent activation of the Bax/caspase-3/PARP-mediated pathway, thus triggering cancer cell death.

## Materials and methods

### Reagents and chemicals

Dulbecco’s modified Eagle’s medium (DMEM), cordycepin (3′-deoxyadenosine, from *C*. *militaris*, C3394), TNF-α (T0157), N_ω_-nitro-l-arginine methyl ester (L-NAME) hydrochloride (CAS 51298-62-5, N5751), and pyrrolidine dithiocarbamate (PDTC, P8765, CAS 5108-96-3) were purchased from Sigma-Aldrich (St. Louis, MO, USA). Fetal bovine serum (FBS), penicillin-streptomycin (10,000 U/ml), and phosphate-buffered saline (PBS) were obtained from Thermo Fisher Scientific (Paisley, Scotland, UK). Annexin-V-FLUOS staining kits were purchased from Roche Diagnostics GmbH (Mannheim, Germany) and Sigma Chemical Co. Bovine serum albumin (BSA) was obtained from Invitrogen Life Technologies (Carlsbad, CA, USA). Whole cell lysis buffer was purchased from Intron Biotechnology Inc. (Seoul, Korea). HilyMax transfection reagent and Cell Counting Kit-8 were purchased from Dojindo Laboratories (Kumamoto, Japan). Dual-Luciferase^®^ reporter assay kit was from Promega (Madison, WI, USA). Antibodies against NF-κB, IκB, GADD45B, MKK7, p-JNK, caspase-3, and β-actin were purchased from Cell Signaling Technology Inc. (Beverly, MA, USA). The JNK inhibitor SP600125 and antibodies against JNK, PARP-1, c-FLIP_L_ (SC-8346), and Bax were purchased from Santa Cruz Biotechnology Inc. (Dallas, TX, USA).

### Cell lines and cell viability assay

Human renal carcinoma cell lines (TK-10 and UO-31) and normal HEK293 cells were obtained from the American Type Culture Collection (Rockville, MD, USA) and cultured in DMEM supplemented with 10% (v/v) FBS and 1% (w/v) penicillin-streptomycin at 37°C in 5% (v/v) CO_2_. Cells were seeded in a 96-well plate (5 × 10^3^ cells/well), incubated for 24 h, and then treated with various concentrations of cordycepin (20, 40, 60, and 80 μg/ml) for 24, 48 and 72 h. A cell viability assay was performed as previously described [18]. Briefly, post treatment, 10 μl of CCK-8 solution was added to each well and the plate was incubated for 1 h at 36°C. Cell viability was determined by measuring the absorbance at 450 nm using a microplate reader (Sunrise^™^, Tecan, Switzerland).

### Cell cycle analysis

To study the apoptotic effect of cordycepin, we analyzed the PI-Annexin-V staining pattern by using the Annexin-V-FLUOS staining kit (Roche Diagnostics). Briefly, cells were treated with cordycepin (20, 40, 60, and 80 μg/ml) for 48 h, collected by scraping, and then washed twice with PBS. The cell suspension was centrifuged at 2,000 rpm for 2 min and incubated with 0.2 mg/ml Annexin-V FLUOS and 1.4 mg/ml PI for 15 min at room temperature. Then, the cells were analyzed on a NucleoCounter^®^ NC-3000^™^ image cytometer (ChemoMetec, Copenhagen, Denmark) using an excitation wavelength of 488 nm, with a 530/30 nm band-pass filter and a 670 nm high-pass filter for detecting Annexin-V and PI, respectively.

### Microarray analysis

The TwinChip^™^ Human-44K microarray (Genocheck, Seoul, Korea) was used for transcript profiling of cordycepin-treated renal cancer cells. Total RNA was extracted from TK-10 cells treated with vehicle or 60 μg/ml cordycepin for 48 h. Genes were considered to be differentially expressed when the global M value, log_2_ (R/G fluorescence), exceeded 1.0 (2-fold change) with a *p*-value < 0.05, after “significance analysis of microarrays.” To analyze the biological significance of the changes, we categorized the microarray data into specific gene groups. The microarray data have been submitted to the Gene Expression Omnibus database (GEO accession numbers: GSE81728)

### Ontology-related network analysis

To study the biological functions of the differentially regulated genes according to ontology-related interaction networks, we conducted a network analysis by using the ingenuity pathway analysis (IPA, http://www.ingenuity.com) tool. Network generation was optimized from the inputted expression profile when possible and was aimed at producing highly connected networks.

### Measurement of NO in TNF-α-stimulated TK-10 cells

The total NO level in cordycepin-treated cells was measured using a modified Griess reaction assay [19]. The NO concentration was estimated indirectly by measuring the concentration of the NO transformation byproduct, nitrite (NO_2_^−^), in living cells. For the measurement of NO/NO_2_^−^, cordycepin-treated TK-10 cells in culture medium were seeded into each well (1 × 10^3^ cells/well) of a 96-well microplate and cultured for 6 h. Aliquots of TNF-α-pretreated TK-10 cells were transferred to the microplate wells in triplicate. After the addition of 50 μl of N1 buffer (1% sulfanilamide in 0.4 N HCl), the pre-reaction was induced for 10 min at room temperature. The final reaction to determine NO_2_^−^ was started by adding 50 μl of N2 buffer (0.1% *N*-(1-naphthyl) ethylenediamine in 0.4 N HCl). After incubating the plate for 10 min at room temperature, differential absorption at 520 and 560 nm was measured using a plate reader. NO_2_^−^ was quantified by comparing the experimental results with those obtained with a standard solution of NaNO_2_. To eliminate the bleaching effect of NADPH, the cell extract was preheated at 30°C for 5 min.

### Luciferase reporter assay

For the NF-κB luciferase assay, TK-10 cells were transfected using the HilyMax reagent according to manufacturer’s instructions. Cells were seeded in a 24-well plate, cultured in DMEM for 18 h prior to transfection, transiently transfected with 3x-κB (NF-κB-Luc plasmid) for 4 h, and incubated overnight. Then, the cells were treated with cordycepin (40, 60, or 80 μg/ml) for 24 h, lysed, and assayed for NF-κB reporter activity using the Dual-Luciferase^®^ kit. Luciferase activity was determined by measuring the luminescence levels of both firefly and Renilla luciferase with a luminometer (Promega). The promoter activity was corrected for transfection efficiency by normalizing to β-galactosidase expression.

### Immunofluorescence microscopy

TK-10 cells were seeded on a coverslip in a 12-well plate (4 × 10^4^ cells/well) and pretreated with cordycepin (60 μg/ml) for 48 h. The cells were incubated overnight with mouse anti-NF-κB p65 IgG1 as a primary antibody (diluted to 1:100 in 3% (w/v) BSA) at 4°C, followed by a 1-h incubation in the dark with fluorescein isothiocyanate-anti-mouse antibody (diluted to 1:200 in 3% (w/v) BSA) as the secondary antibody. The coverslips were washed with PBS and mounted with mounting solution for observation. Images were acquired using an LSM 710 laser-scanning confocal microscope (Carl Zeiss, Jena, Germany) equipped with a C-Apochromat 40×/1.2 water immersion lens (488 nm Ar laser/505–550 nm detection range) and analyzed using the ZEN 2009 Light Edition software (Carl Zeiss).

### Gene knockdown

The c-FLIP_L_ plasmid construct, pGL3-FLIP1500 (Addgene plasmid #16016), was a gift from Wafik El-Deiry [20]. siRNAs for GADD45B, MKK7, and c-FLIP_L_ with the sequences, 5′-CGU UCU GCU GCG ACA AUG A-3′, 5′-GCA UUG AGA UUG ACC AGA A-3′, and 5′-AAC ATC CAC AGA ATA GAC CTG CCT GTC TC-3′, respectively, were purchased from ST Pharm (Seoul, Korea). TK-10 cells were seeded in 6-well plates (2 × 10^5^ cells/well). After incubation, the cells were supplied with growth medium containing 10% FBS and harvested 48 h later for siRNA transfection, using Lipofectamine^®^ RNAiMAX reagent (Invitrogen, Carlsbad, CA, USA) per the manufacturer’s instructions. Then, the cells were treated with cordycepin (60 μg/ml) for 48 h.

### Western blot analysis

The expression of apoptosis-related signaling proteins in whole lysate or cytosolic and nuclear fractions from cordycepin-treated cells was examined by western blotting, as described previously [21]. In brief, denatured protein (30 μg) was separated on a 12% polyacrylamide gel and transferred onto a nitrocellulose membrane. The blotted membrane was blocked for 1 h with 5% (w/v) skimmed milk in TTBS (Tween-20 and Tris-buffered saline (TBS)). The membrane was incubated at room temperature for 2 h or overnight with primary antibodies against the following antigens (at the indicated dilutions): NF-κB (1:1000), IκB (1:1000), GADD45B (1:500), JNK (1:200), p-JNK (1:200), MKK7 (1:500), caspase-3 (1:500), Bax (1:1000), PARP (1:1000), c-FLIP_L_ (1:200), and β-actin (1:2000). The membrane was washed with TTBS thrice for 5 min each time and incubated with horseradish-peroxidase (HRP)-conjugated goat anti-mouse IgG or HRP-conjugated rabbit anti-goat IgG (1:2000 dilution in TBS containing 5% (w/v) skimmed milk) at room temperature for 1 h. The bands were visualized on a ChemiDoc^™^ MP system (Bio-Rad, Hercules, CA, USA) using an enhanced chemiluminescence system (Thermo Scientific, San Jose, CA, USA), and densitometric measurements were conducted using the ImageJ software (National Institutes of Health, Bethesda, Maryland, USA).

### Wound-healing assay

Both control and cordycepin-treated cells were subjected to a wound-healing assay to study the effect of cordycepin. Cell monolayers in 24-well plates were scraped in a straight line with a p200 pipette tip to create a wound and then treated with cordycepin (60 μg/ml) for 48 hr. Cell migration was analyzed using the TissueFAXS system (TissueGontics, Vienna, Austria) and area, number, and grade were quantified using the HistoQuest software (TissueGnostics).

### Tumor xenograft experiment

For tumor xenograft experiments, 5-week-old male nude mice (nu/nu BALB/c) were purchased from SLC, Inc. (Hamamatsu, Japan). There were 6–8 PBS-treated control, and 6–8 20 mg/kg cordycepin treated mice used in each experimental group. Mice were housed (3–4/cage) with food and water available *ad libitum*. The protocol was adapted from studies in our and other laboratories [22]. Mice were injected subcutaneously into the hind limb with TK-10 cells (1 × 10^6^ cells/ml) suspended in DMEM for 5 day and check the volumes of tumor size. Tumors were calculated by three-dimensional ultrasonography (Philips IU22 Ultrasound, KPI Ultrsound Inc., Yorba Linda, CA, USA) every 2 days. When tumors reached a mean size of 0.2–0.3 cm3, the animals were divided into groups with comparable tumor size and treated as described in the text and figures. To determine the cordycepin effects, the mice were then treated with PBS (n = 6) or 20 mg/kg cordycepin (n = 6) once daily for 20 days and maintained in Association for Assessment and Accreditation of Laboratory Animal Care-approved barrier facilities on a 12-h light-dark cycle, with *ad libitum* access to food and water. Mice should be euthanized, when the volume of the tumor reaches 1/5 of the total volume of the body. However, the tumor volume did not reach the threshold, so we did not sacrificed mice until the completion of our study. Mice should be euthanized when their weight reaches a third of their average weight, but no animal was sacrificed due to this criteria during our study. The mice were sacrificed with isoflurane at 21 days post injection. The tumors were collected and fixed with 4% paraformaldehyde in PBS. The animal study was conducted in accordance with ethical guidelines and reviewed and approved by the Institutional Animal Care and Use Committee (IACUC) of the Korea Basic Science Institute (KBSI, KBSI-ACE-022-2015).

### Immunohistochemistry

Five-millimeter-thick sections were cut across the dorsoventral diameter of the tumors, fixed in ice-cold 10% paraformalin overnight, and embedded in paraffin. Serial sections (4 μm thick) were cut and processed for immunohistological staining. The slides were quenched in 3% hydrogen peroxide to block endogenous peroxidase activity, washed in TBS (0.05 M, pH 7.6), and blocked with 3% BSA in PBS for 1 h at room temperature. The slides were incubated overnight at 4°C with primary antibodies (diluted in serum-free protein-blocking buffer) directed against the following antigens (at the indicated dilutions): MKK7 (1:500; Dako, Glostrup, Denmark), c-FLIP_L_ (1:200; Santa Cruz Biotechnology Inc.), and JNK (1:200, Santa Cruz Biotechnology Inc.). A labeled streptavidin-biotin kit (Dako) was used for the avidin-biotin-peroxidase complex method, and the slides were counterstained with hematoxylin. Finally, the slides were dehydrated in an ethanol series, rinsed with xylene, and mounted with Permount.

### Statistical analysis

Student’s *t*-test was used to assess differences between control and cordycepin-treated groups, using the GraphPad Prism software (GraphPad, San Diego, CA, USA). *p* < 0.05 was considered statistically significant.

## Results

### Cordycepin induces apoptotic changes in renal cancer cells

**Fig 1:**
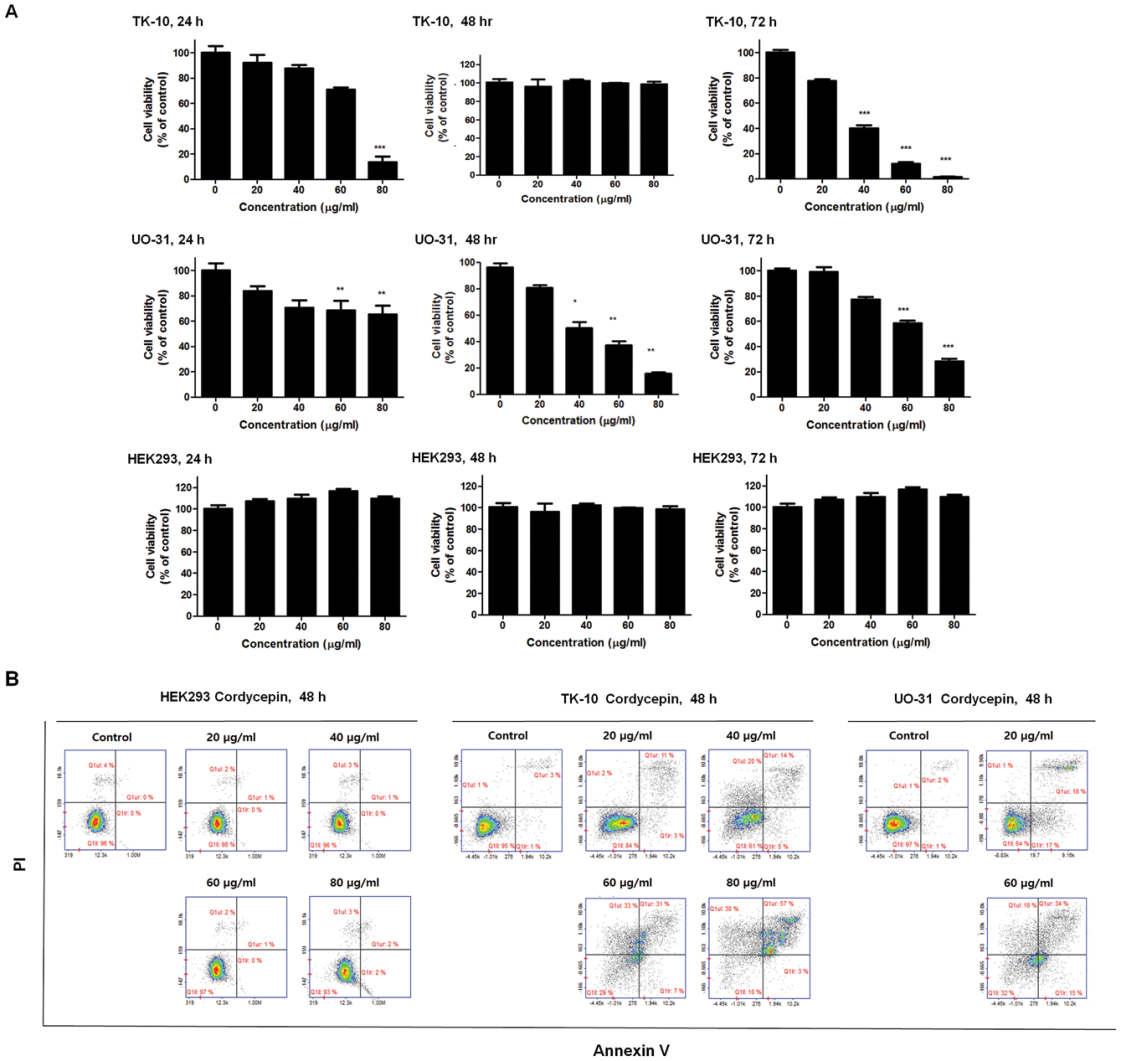
Cordycepin induces apoptosis in renal cancer cells. (A) Inhibition of renal cancer cell growth by cordycepin. TK-10 and UO-31 cancer cells were treated with the indicated concentrations of cordycepin for 24, 48 and 72 h. HEK293, Human embryonic kidney cell line was used as normal cells. Data are presented as the mean ± standard deviation (SD) from triplicate experiments. *p < 0.05 and ** *p* < 0.01 vs. untreated control (B) Flow-cytometric analysis of TK-10, UO-31 and HEK293 cells post cordycepin treatment stained with PI and Annexin-V. The data are representative of three independent experiments.

The effects of cordycepin on cancer cell proliferation were investigated by treating TK-10, UO-31 and HEK293 cells with various concentrations of cordycepin (0, 20, 40, 60, and 80 μg/ml) for 24, 48, 72 h. Cordycepin dose-dependently inhibited TK-10 and UO-31 cell growth during the 48-h incubation period, whereas the effects of cordycepin on viability were increased in 72 hr treatment in the UO-31 cell viability. (Fig 1A). Therefore, the apoptotic effect of cordycepin was determined to be 60 μg/ml for 48 h (Fig 1A). Apparently, Cordycepin 0, 20, 40, 60, and 80 μg/ml had no effect on proliferation of HEK293.

The apoptotic effect of cordycepin was analyzed by flow cytometry using Annexin V- and PI-stained TK-10 and UO-31 cells after 48-h treatment with cordycepin (20, 40, 60, and 80 μg/ml). While 20 μg/ml cordycepin caused no drastic change in the Annexin V-stained viable fraction of TK-10 cells (95% to 84%), 40 μg/ml cordycepin shifted the cells to the late apoptotic stage (3% to 14%). Similarly, treatment with 40 μg/ml cordycepin shifted UO-31 cells to the late apoptotic stage (2% to 18%). There was a marked shift from the normal to the late apoptotic stage in TK-10 (3% to 31%) and UO-31 (2% to 34%) cells upon treatment with 60 μg/ml of cordycepin, with a reduction in the viable fraction from 95% to 29% (TK-10) and 97% to 32% (UO-31) (Fig 1B). In contrast apoptotic change of HEK293 was not observed (0% to 2.00%). Thus, treatment with 60 μg/ml of cordycepin induced renal cancer cell apoptosis.

### Effects of cordycepin on gene expression

To identify the genes involved in the antitumor activity of cordycepin, microarray analysis of cordycepin-treated (60 μg/ml) TK-10 cells was conducted. Among the 63,242 unique genes assayed, 28,858 genes were expressed in cordycepin-treated cells. Among these 28,858 genes, cordycepin treatment upregulated and downregulated 1,461 and 942 genes, respectively, in comparison to the levels observed in the untreated control, at 48 h. Genes that were up- or downregulated by more than 2-fold were handled as significant in data-mining categories.

**Fig 2:**
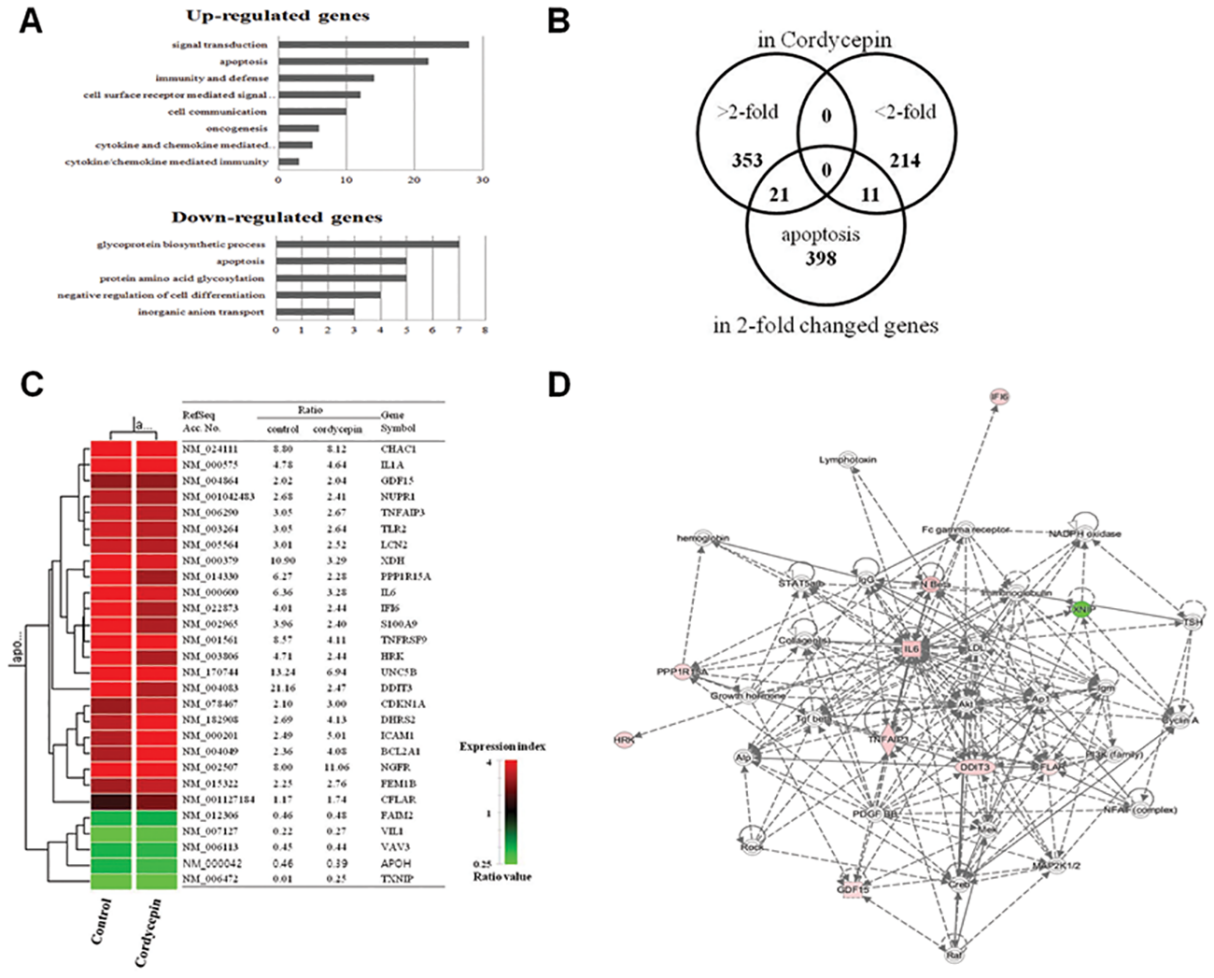
Cordycepin induces genes involved in inflammation and apoptosis. (A) Gene ontology analysis of genes differentially expressed in cordycepin-treated (60 μg/ml) TK-10 cells versus untreated cells. (B) Gene lists (>2-fold, <2-fold, and apoptosis-related genes) and Venn diagrams of the numbers of genes up- and downregulated in each experimental group. (C) Hierarchical clustering of apoptotic genes deregulated in response to cordycepin. (D) Signaling network of the apoptotic genes in response to cordycepin. Nodes colored using IPA are the genes in the apoptotic regulatory network in cordycepin-treated TK-10 cells (red: upregulated genes, green: downregulated genes).

Biologically relevant features were constructed using the Database for Annotation, Visualization, and Integrated Discovery (DAVID) tools (http://david.abcc.ncifcrf.gov/). Lists of 2-fold upregulated and downregulated genes in cordycepin-treated renal cancer cells were uploaded to DAVID for gene ontology analysis (Fig 2A). Upregulated genes included those involved in signal transduction, apoptosis, immunity and defense, cell-surface receptor-mediated signaling, cell communication, oncogenesis, and cytokine- and chemokine-mediated signaling and immunity. Downregulated genes included those involved in glycoprotein biosynthesis, apoptosis, protein glycosylation, negative regulation of cell proliferation, and inorganic anion transport. The genes deregulated by cordycepin treatment were compared with potential apoptotic genes by identifying candidate genes using the GeneCards database (http://www.genecards.org/) (Fig 2B). The intersection obtained by hierarchical clustering is presented along with the gene lists in Fig 2C. The signaling network of cordycepin-responsive apoptotic genes is shown in Fig 2D.

### Cordycepin inhibits the activation and promoter activity of NF-κB

**Fig 3:**
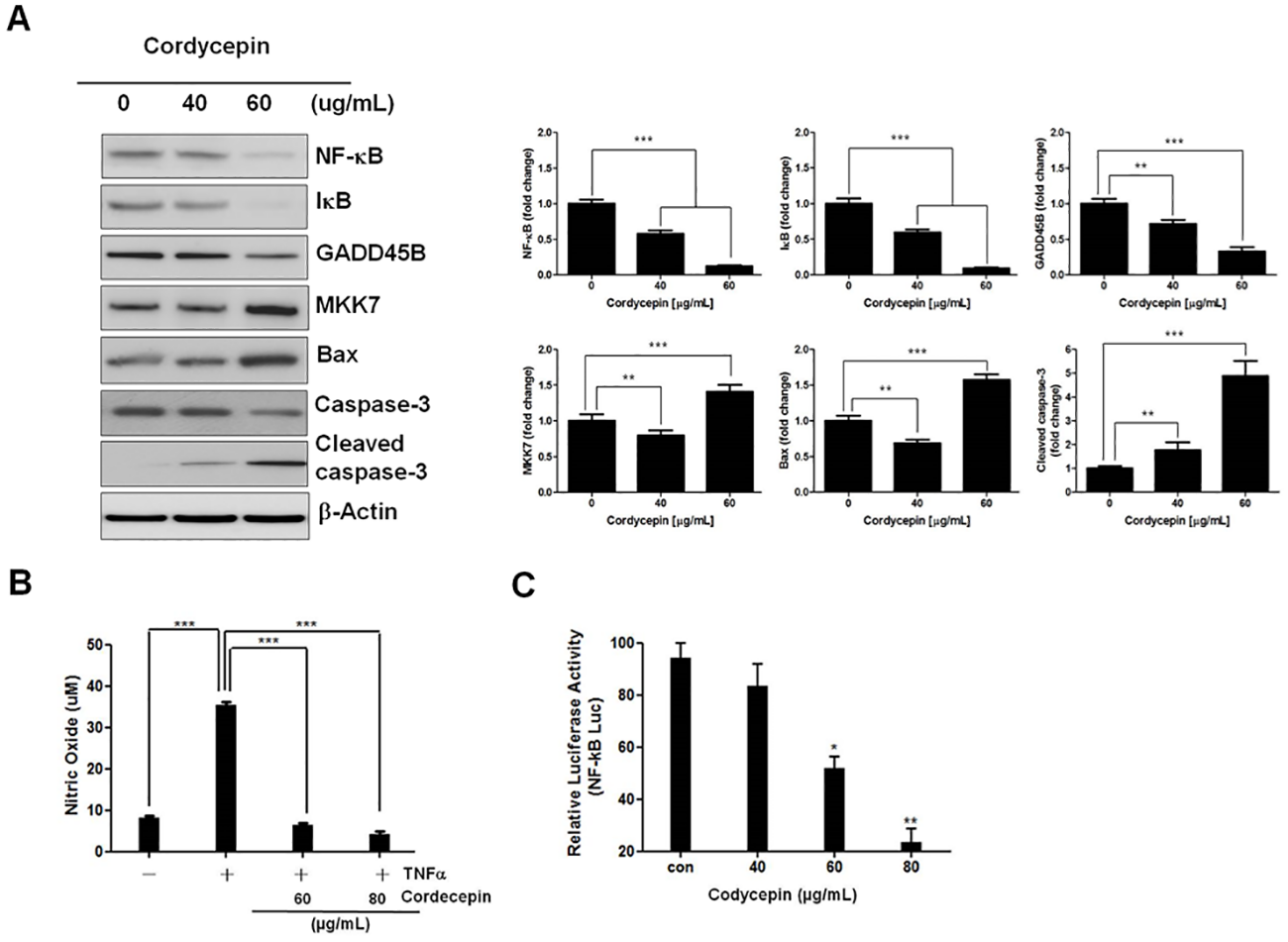
Cordycepin inhibits the IκB/NF-κB signaling pathway. (A) Effect of NF-κB on GADD45B and MKK7 expression in cordycepin-treated TK-10 cells. (B) Effect of cordycepin on NO production in TNF-α-stimulated TK-10 cells. Each column represents the mean ± SD of three independent experiments. (C) Estimation of NF-κB promoter activity in TK-10 cells by luciferase assay. Luciferase activity was determined by measuring the luminescence levels of both firefly and Renilla luciferase with a luminometer. **p < 0.01 and *** *p* < 0.001.

To investigate whether cordycepin inhibits NF-κB activation, western blotting was used to detect the expression of IκB, NF-κB, GADD45B, MKK7, Bax, caspase-3, and cleaved caspase-3 in TK-10 cells treated with 40 or 60 μg/ml of cordycepin. Cordycepin reduced IκB, NF-κB, GADD45B, and increased MKK7, Bax, and cleaved caspase-3 levels dose-dependently (Fig 3A). NF-κB is a transcription factor that plays a crucial role in cytokine- and TNF-α-induced gene activation during inflammatory events [23]. Incubation of TK-10 cells with NO donors inhibits TNF-α-induced NF-κB activation by inducing and stabilizing the NF-κB inhibitor, IκB. As constitutively produced NO levels in TK-10 cells were at the lower sensitivity limit of the assay, the cells were stimulated with TNF-α (20 ng/ml) for 24 h to induce NO production.

Cordycepin significantly decreased TNF-α-induced NO production in a dose-dependent manner (60–80 μg/ml cordycepin) (Fig 3B), suggesting that NF-κB activation is clearly inhibited by cordycepin. NF-κB promoter activity was measured by luminometry after a 4-h transfection of TK-10 cells with the 3x-κB plasmid. Cordycepin treatment (40, 60, and 80 μg/ml) significantly decreased NF-κB activity to 12%, 52%, and 76% of the control, respectively (Fig 3C), suggesting that it blocks the normal signal transduction to transcription factors such as NF-κB.

### Cordycepin inhibits activation of the NO-mediated NF-κB signaling pathway

**Fig 4:**
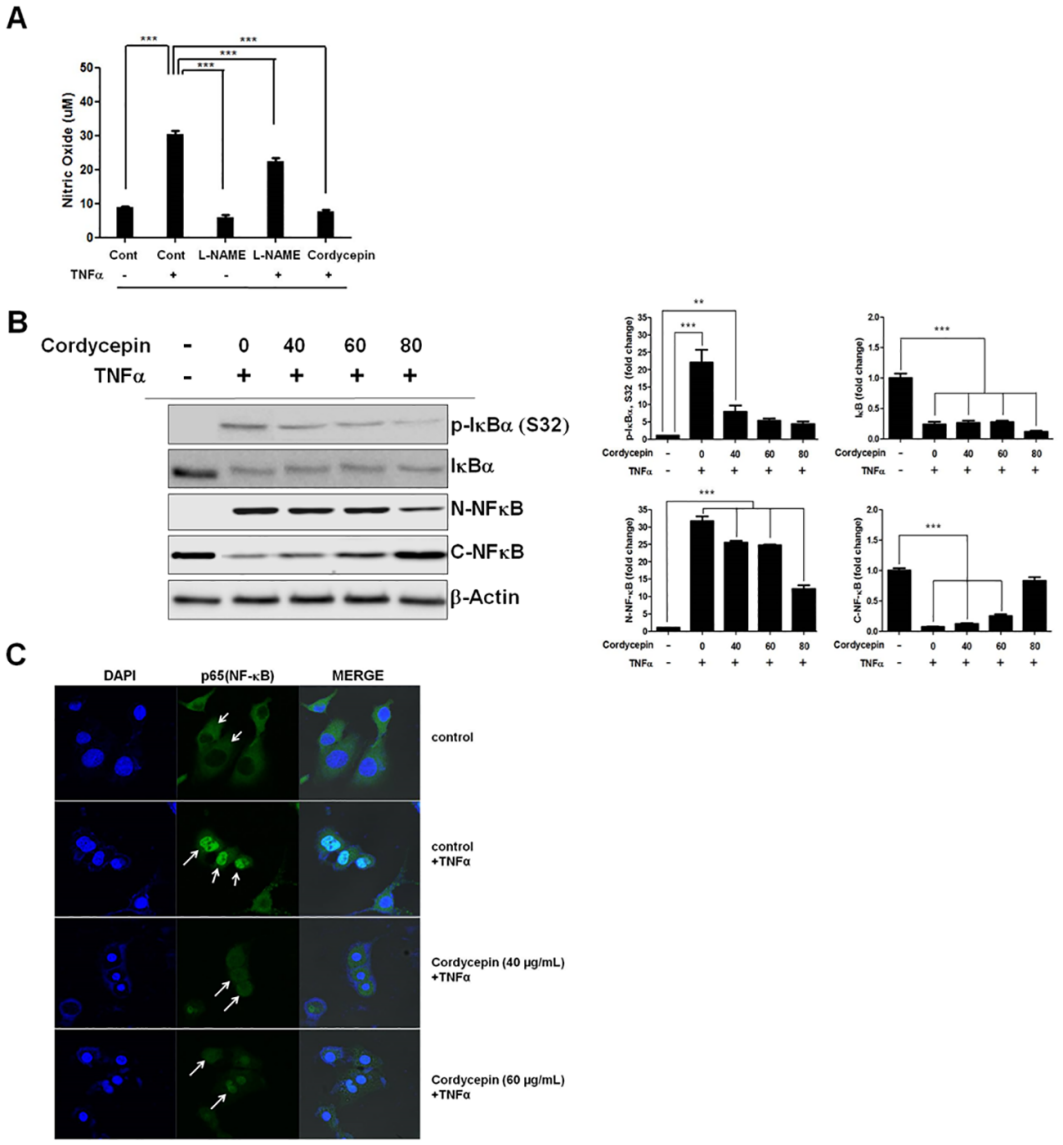
Cordycepin activates the NF-κB pathway in TNF-α-stimulated TK-10 cells. (A) Inhibition of TNF-α-induced NO production by cordycepin. (B) Effect of cordycepin on IκB-α (S32) phosphorylation in TNF-α-treated TK-10 cells. The data represent the mean ± SE from three independent experiments. **p < 0.01 and *** *p* < 0.001 (C) Immunofluorescence micrographs of NF-κB (p65) translocation in TK-10 cells. White arrows indicate p65 retained in the cytoplasm. The data represent the mean ± SE from three independent experiments. **p < 0.01 and *** *p* < 0.001.

The NO level in supernatants of cordycepin-treated TK-10 cells was estimated to determine whether cordycepin exerts apoptotic activity by inhibiting NO (Fig 4A). Treatment of the TK-10 cells with L-NAME (500 μM), a well-known NO synthase inhibitor, for 24 h resulted in decreased NO production, as compared to that observed in untreated control cells. When L-NAME-treated cells were further treated with TNF-α (20 ng/ml) for 24 h, the L-NAME-induced inhibition of NO production was blocked by TNF-α, which also increased the NO production in comparison to that observed in untreated control cells. These results indicated that endogenous NO plays a pivotal role in TK-10 cell proliferation.

Next, we analyzed the effect of TNF-α on the cordycepin-induced inhibition of NO production by TK-10 cells treated with TNF-α for 24 h. Cordycepin (60 μg/m) treatment blocked the TNF-α stimulated NO production (Fig 4A). NF-κB is a critical transcription factor regulating inflammation-related gene expression in TNF-α-activated TK-10 cells. It is usually sequestered in the cytoplasm as an inactive precursor complex by its inhibitory protein, IκB. After stimulation, IκB is phosphorylated by IκB kinase, ubiquitinated, and rapidly degraded through proteasomes. This releases the p65 subunit of NF-κB, which translocates to the nucleus and activates numerous genes. We found that cordycepin significantly reduced IκB-α (S32) phosphorylation in TNF-α-treated TK-10 cells (Fig 4B), leading to p65 accumulation in the cytoplasm and reduced nuclear p65 levels (Fig 4C). These results indicated that cordycepin can at least attenuate NF-κB signaling in TNF-α treated-TK-10 cells, and thus inhibit the NO-mediated IκB/NF-κB inflammatory response.

### Cordycepin promotes MKK7-JNK signaling by suppressing NF-κB-induced c-FLIP_L_ expression

**Fig 5:**
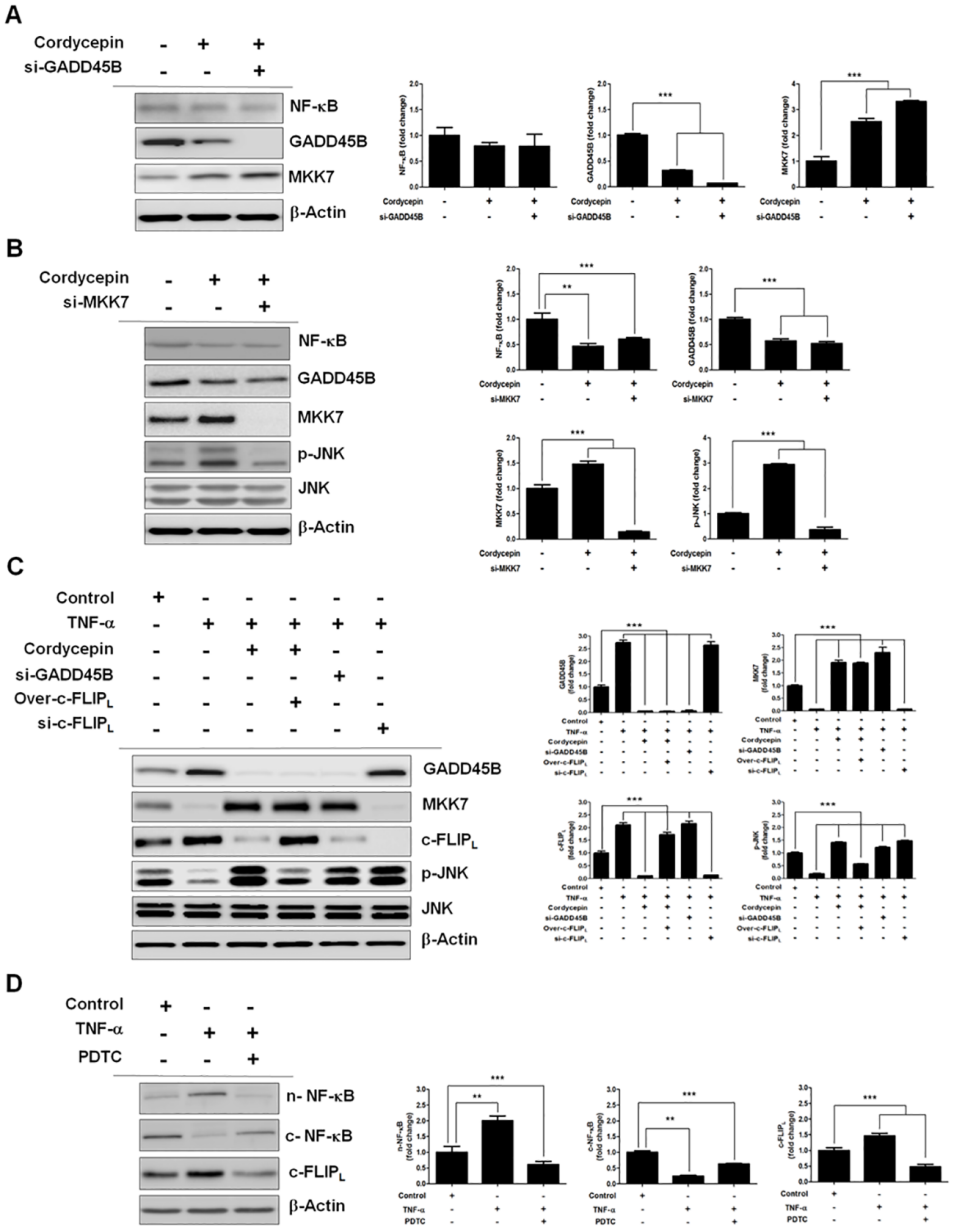
Cordycepin mediates the regulation of MKK7 by GADD45B via NF-κB suppression. (A) Effect of GADD45B on MKK7 expression in cordycepin-treated TK-10 cells. (B) Effect of MKK7 on JNK expression in cordycepin-treated TK-10 cells. (C) Effect of TNF-α-induced NF-κB on c-FLIP_L_ expression. (D) Effect of c-FLIP_L_ on JNK expression in cordycepin-treated TK-10 cells. The data represent the mean ± SE from three independent experiments. **p < 0.01 and *** *p* < 0.001.

NF-κB is an important upstream mediator in the MKK7 signaling pathway. Therefore, we investigated whether cordycepin promotes MKK7 activation by suppressing NF-κB, and the effect of GADD45B on MKK7, in cordycepin-treated TK-10 cells. While cordycepin downregulated NF-κB and GADD45B, MKK7 expression was upregulated. siRNA-mediated knockdown of GADD45B clearly increased MKK7 expression as compared to cordycepin treatment alone, without affecting NF-κB expression (Fig 5A). These results indicated that cordycepin mediates the upregulation of MKK7 by inhibiting GADD45B expression. Moreover, GADD45B inhibits apoptosis by binding to MKK7, thus suppressing JNK signaling. Hence, we investigated whether GADD45B mediates an anti-apoptotic crosstalk between the NF-κB and JNK pathways (Fig 5B) by observing the effect of MKK7 on JNK in cordycepin-treated TK-10 cells. While cordycepin downregulated NF-κB and GADD45B, the expression of MKK7 and p-JNK was upregulated. Further, siRNA-mediated knockdown of MKK7 decreased p-JNK expression, without affecting NF-κB and GADD45B expression. These results indicated that cordycepin mediates the regulation of JNK by MKK7 via the IκB/NF-κB/GADD45B signaling pathway.

Next, we analyzed the effect of NF-κB on c-FLIP_L_ expression in TK-10 cells treated with TNF-α for 24 h (Fig 5C). TNF-α promoted NF-κB translocation to the nucleus, which resulted in increased c-FLIP_L_ expression, whereas PDTC, a potent NF-κB inhibitor, inhibited nuclear translocation of NF-κB, resulting in the downregulation of c-FLIP_L_ expression. Additionally, we analyzed the effect of c-FLIP_L_ on JNK activation in the TNF-α-treated TK-10 cells. TNF-α increased c-FLIP_L_ expression, whereas cordycepin decreased TNF-α-induced c-FLIP_L_ expression. siRNA-mediated knockdown of c-FLIP_L_ prevented TNF-α-induced JNK inactivation, whereas c-FLIP_L_ overexpression inhibited cordycepin-mediated JNK activation without affecting GADD45B and MKK7 expression. Furthermore, siRNA-mediated inhibition of GADD45B upregulated JNK activation (p-JNK) without affecting c-FLIP_L_ expression (Fig 5D).

### Cordycepin enhances JNK-mediated Bax activation

**Fig 6:**
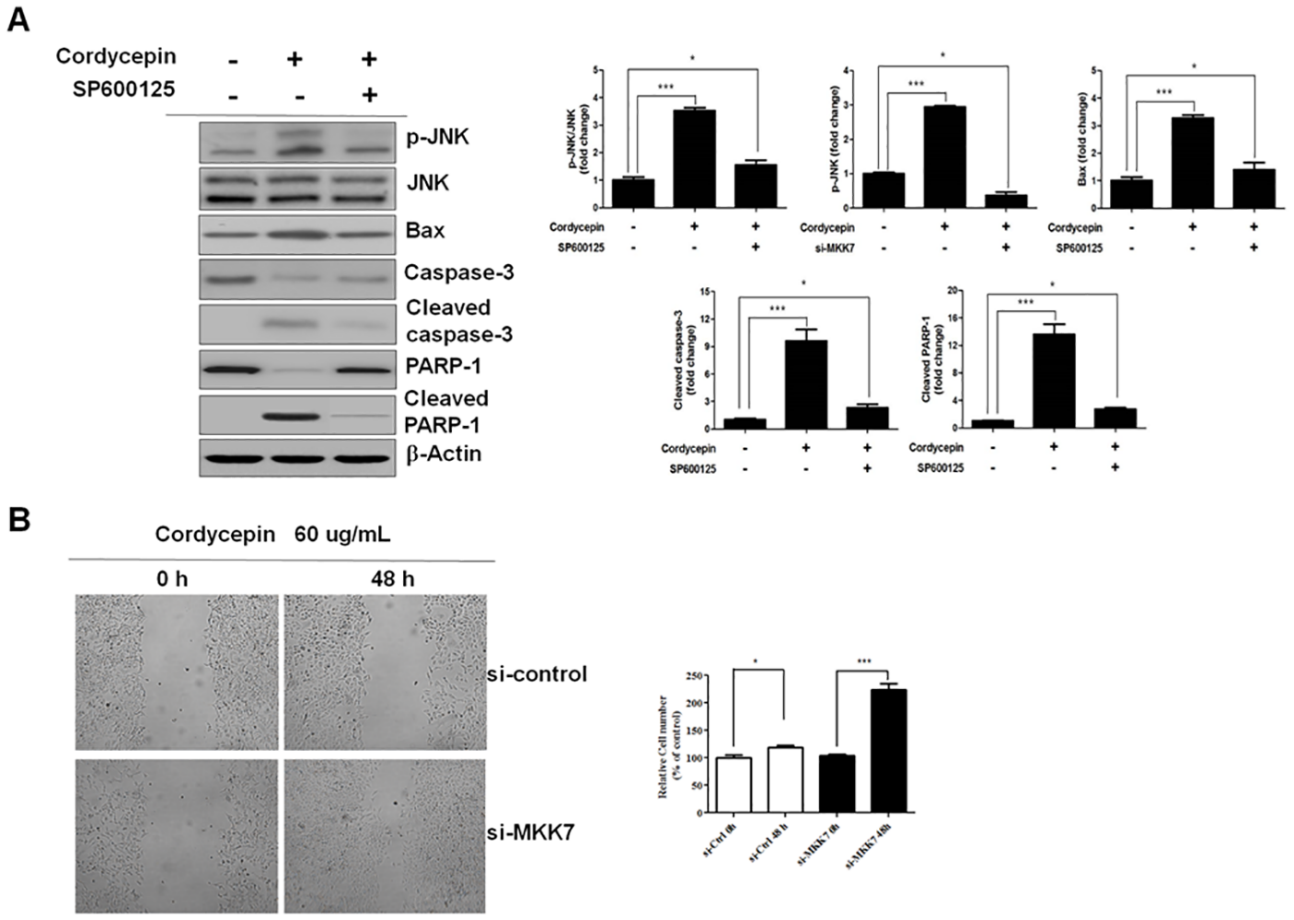
Cordycepin enhances JNK-mediated Bax activation and reduces tumor cell migration. (A) Effect of p-JNK on Bax expression in cordycepin-treated TK-10 cells. (B) Effects of treatment with cordycepin or a combination of cordycepin and MKK7 siRNA on TK-10 cell migration. Migrating cells were photographed under an inverted microscope (magnification 100×). The migration data are presented as the mean ± SD of three independent experiments performed in triplicate. *p < 0.05 and ***p < 0.001, vs. the control (0 h).

There exists multilevel crosstalk between the pro-apoptotic JNK and pro-survival GADD45B pathways. Therefore, we examined Bax protein expression after cordycepin treatment to determine whether JNK-mediated regulation of Bax is related to NF-κB/GADD45B signaling. Western blotting was used to detect the expression of JNK, p-JNK, Bax, caspase-3, cleaved caspase-3, PARP-1, and cleaved PARP-1, and to determine the effects of the JNK-specific inhibitor, SP600125, on Bax, in TK-10 cells treated with 60 μg/ml cordycepin. While cordycepin was observed to upregulate the expression of p-JNK, Bax, cleaved caspase-3, and cleaved PARP-1, SP600125 downregulated their expression (Fig 6A). These results indicated that cordycepin induces JNK/Bax signaling-mediated apoptosis by inhibiting the NF-κB/GADD45B signaling pathway.

The biological relevance of the regulation of MKK7 was evaluated by testing its effect on directed migration of TK-10 cells by a wound-healing assay. While TK-10 migration was significantly suppressed by cordycepin (*p* < 0.05), MKK7 silencing significantly recovered the migration (Fig 6B).

### Upregulation of MKK7 and p-JNK correlates with decreased survival in renal cancer cells

**Fig 7:**
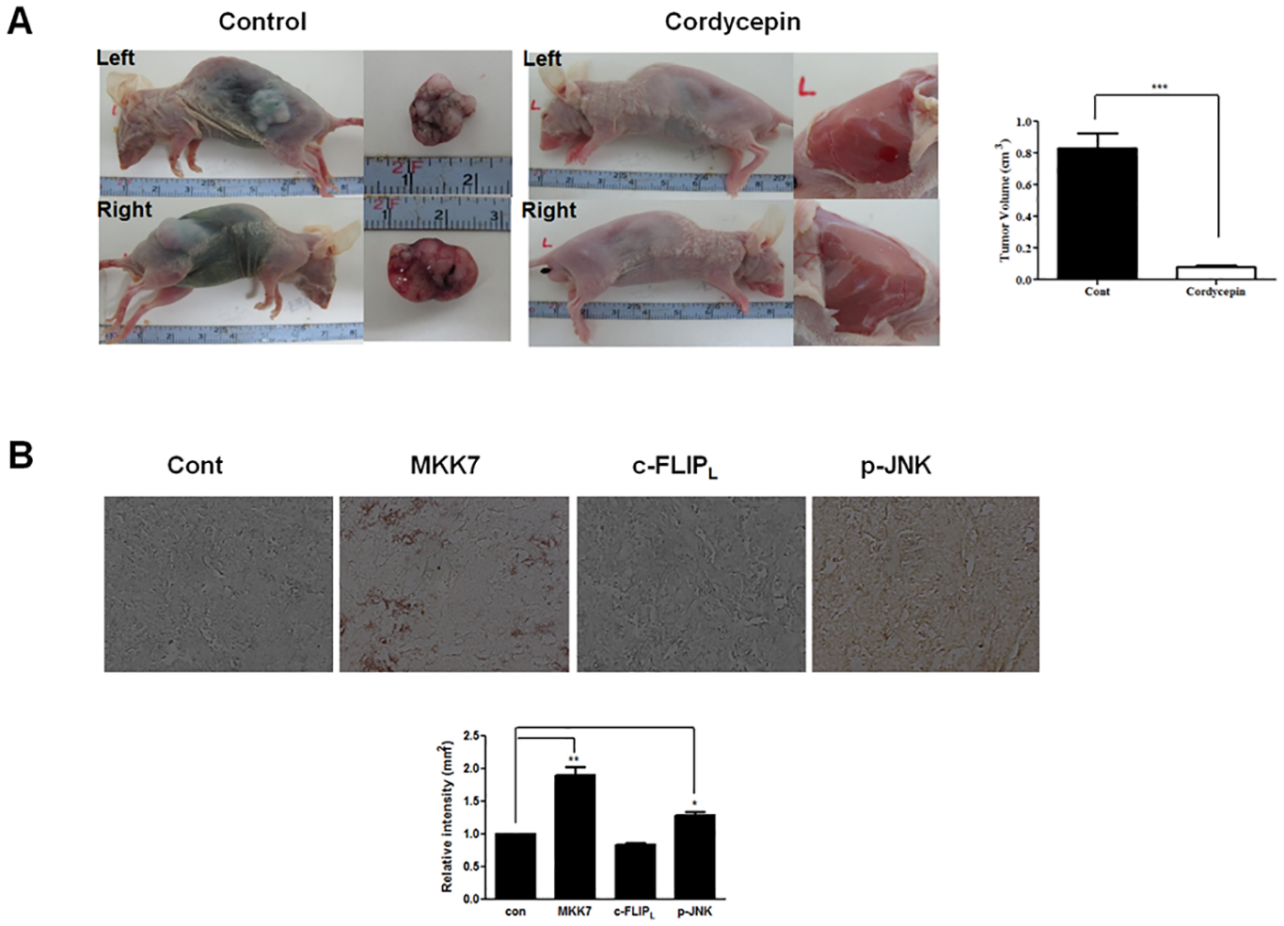
Effect of cordycepin on the expression of MKK7, c-FLIP_L_, and p-JNK in renal tumor tissues. (A) Cordycepin induces tumor growth suppression. Nude mice were injected with TK-10 cells and then treated with either PBS or 20 mg/kg cordycepin once daily for 20 days. Representative dissected tumors are shown on the right. (B) Immunohistochemical analysis of tumor tissues. Data are the mean ± SD of three independent experiments performed in triplicate. *p < 0.05, ** *p* < 0.01.

Since cordycepin upregulated MKK7 expression and JNK phosphorylation *in vitro*, we examined the *in vivo* expression of MKK7, c-FLIP_L_, and p-JNK in tumor tissues by immunohistochemistry. We treated with cordycepin for 20 days when the mean tumor volume reached 0.2 cm^3^. We identified tumor volume using ultrasound equipment and the maximum volume was 0.64 cm^3^ when cordycepin was not treated and did not change significantly when weighing every two days (S1 Table). Tumors were calculated by three-dimensional ultrasonography every 2 days (S1 Table). In comparison to untreated controls, mice treated with cordycepin showed significantly suppressed tumor growth (Fig 7A) as well as upregulated MKK7 expression and JNK phosphorylation, and downregulated c-FLIP_L_ expression (Fig 7B). These results suggested that cordycepin can arrest tumor cell growth *in vivo*. Thus, cordycepin was shown to effectively inhibit tumor growth both *in vitro* and *in vivo*.

## Discussion

Cordycepin has been reported to exert antitumor, antiviral, anti-oxidant, and anti-inflammatory activities [24,25,26,27]. Several recent reports suggested that cordycepin exerts its anticancer and antimetastatic effects by inhibiting the expression of some inflammatory genes [28] and some molecules critically involved in tumor growth and metastasis [29,30], by suppressing NF-κB. In the present study, we showed that the treatment of human renal cancer cells with cordycepin inhibited TNF-α-mediated NF-κB/GADD45B signaling, which upregulated the MKK7-JNK signaling pathway activation through inhibition of c-FLIP_L_ expression. First, we found that cordycepin inhibited TNF-α-induced NF-κB/GADD45B signaling, which activated the MKK7-JNK signaling. Second, cordycepin inhibited NF-κB-mediated c-FLIP_L_ activation. Third, knockdown of c-FLIP_L_ prevents TNF-α-induced JNK inactivation. Finally, cordycepin activates MKK7-JNK signaling through inhibition of c-FLIP_L_ expression and, consequently, induces apoptosis.

Aberrant upregulation and activation of pro-inflammatory cytokines and chemokines, including TNF-α, IL-1β, IL-6, IL-8, CCL5, and CXCL8, has been correlated with the progression of chronic inflammatory diseases, such as tumor, sepsis, rheumatoid arthritis, psoriasis, and cytotoxicity [31,32,33]. Further, NO regulates NF-κB activation and facilitates its translocation into the nucleus by exogenous NO donors in TNF-α-activated vascular endothelial cells [34]. Therefore, we investigated whether cordycepin inhibits the NO-mediated NF-κB signaling by estimating the NO level in TNF-α- and cordycepin-treated TK-10 cells using ELISA. The elevated NO level in TNF-α-treated TK-10 cells was downregulated by subsequent treatment with cordycepin (Fig 4A). Moreover, cordycepin downregulated IκB-α (S32) phosphorylation in TNF-α-treated TK-10 cells (Fig 4B), which resulted in cytoplasmic accumulation of NF-κB (p65) and thus, reduced nuclear p65 level. These results indicate that cordycepin inhibits NF-κB signaling in TNF-α-treated TK-10 cells and thus, can efficiently inhibit the NO-mediated IκB/NF-κB inflammatory response. NF-κB signaling promotes survival in cancers. The interaction between NF-κB-regulated GADD45B and MKK7 has been identified as a potential therapeutic target in multiple myeloma [17]. Similarly, we identified GADD45B/MKK7 signaling, which occurs downstream of NF-κB, to be a potential therapeutic target in TK-10 cells (Fig 5). Furthermore, cordycepin was shown to effectively induce MKK7-JNK-dependent apoptosis in TK-10 cells (Fig 6A). Our study revealed the precise mechanism by which cordycepin suppresses GADD45B-mediated MKK7 inhibition by downregulating NF-κB [35]. Our findings also uncovered a mechanism for the pathogenic survival activity of NF-κB in TK-10 cells. We showed that cordycepin promotes MKK7 activation via NF-κB suppression, and the inhibitory effect of GADD45B on MKK7, in cordycepin-treated TK-10 cells. Cordycepin downregulated NF-κB activation and GADD45B expression (Figs 4B and 5A) and upregulated MKK7 and p-JNK expression (Fig 5A and 5B). siRNA-mediated knockdown of GADD45B after cordycepin treatment markedly increased MKK7 expression as compared to cordycepin alone, without affecting NF-κB expression (Fig 5A). These results indicate that cordycepin mediates the upregulation of MKK7 expression by inhibiting GADD45B activation via NF-κB downregulation.

We also investigated whether MKK7 mediated an apoptotic crosstalk between the NF-κB and JNK signaling pathways by observing the effect of MKK7 on JNK in cordycepin-treated TK-10 cells. Knockdown of MKK7 inhibited p-JNK, but did not change NF-κB and GADD45B expression (Fig 5B). In previous studies, NF-κB signals induced the expression of c-FLIP [36], and downregulation of c-Flip expression upregulated caspase-dependent JNK activation and reactive oxygen species accumulation in cancer cells [37]. We found that TNF-α promoted NF-κB translocation to the nucleus, which resulted in the upregulation of c-FLIP_L_ expression, whereas PDTC inhibited NF-κB nuclear translocation, which resulted in the downregulation of c-FLIP_L_ expression (Fig 5C). These results indicate that c-FLIP_L_ expression can be regulated by TNF-α-induced NF-κB signaling. Additionally, we found that cordycepin decreased TNF-α-induced c-FLIP_L_ expression, and that knockdown of c-FLIP_L_ prevented TNF-α-induced JNK inactivation, whereas c-FLIP_L_ overexpression inhibited cordycepin-mediated JNK activation (Fig 5D). These results indicate that cordycepin upregulates MKK7-JNK signaling by inhibiting c-FLIP_L_. Generally, JNK activation is associated with apoptosis induction, whereas NF-κB activation protects against TNF-α-induced apoptosis by suppressing the JNK cascade. Therefore, we evaluated the fundamental role of NF-κB in JNK signaling regulation in cordycepin-treated TK-10 cells. The levels of p-JNK and Bax proteins were dramatically increased in cordycepin-treated TK-10 cells, while NF-κB activation was decreased. The role of JNK in cordycepin-induced apoptosis was further investigated using the JNK inhibitor, SP600125 (Fig 6A). SP600125 treatment decreased the Bax, cleaved caspase-3, and cleaved PARP-1 levels in cordycepin-treated TK-10 cells. This suggests that Bax is activated owing to JNK activation by cordycepin-induced NF-κB inhibition. Collectively, these results indicate that cordycepin mediates the apoptotic regulation of MKK7-JNK signaling by inhibition of c-FLIP_L_ expression through the downregulation of TNF-α induced NF-κB activation. This implies that cordycepin can regulate aspects of transcriptional responses. In conclusion, our systematic investigation revealed the detailed molecular mechanism underlying cordycepin-induced JNK signaling activation through inhibition of c-FLIP_L_ expression, and demonstrates the potential of cordycepin as a therapeutic agent for renal cancer treatment.

## Supporting information

S1 Table Animal health monitoring with maximum size of tumor. TK-10 cells were xenografted to the left and right thighs of nude mice and treated with cordycepin for 20 days at a mean tumor volume of 0.2 cm^3^. The volume of the tumor was determined on a 2-day interval using three-dimensional ultrasonography (Philips IU22 Ultrasound). The maximum volume for no treated with codycepin was 0.64 cm^3^ on the right. When treated with cordycepin, both side were significantly reduced to 0.001 cm^3^. There was no significant change in body weight when measured at 2-day intervals.
(DOC)

## Abbreviations

DAVID: Database for Annotation, Visualization, and Integrated Discovery
ERK: extracellular signal-regulated kinase
FLIP: FLICE-inhibitory protein
GADD45B: growth arrest and DNA damage-inducible beta
HRP: horseradish-peroxidase
IPA: ingenuity pathway analysis
JNK: Jun N-terminal kinase
JNKK2: JNK kinase 2
L-NAME: N_ω_-nitro-l-arginine methyl ester
LPS: lipopolysaccharide
MAPK: major mitogen-activated protein kinase
MKK7: mitogen-activated protein kinase kinase 7
NO: nitric oxide
PI: propidium iodide
RNS: reactive nitrogen species
TNFα: tumor necrosis factor alpha, Tween-20 and Tris-buffered saline
