# Correction: Cordycepin promotes apoptosis in renal carcinoma cells by activating the MKK7-JNK signaling pathway through inhibition of c-FLIP_L_ expression

**DOI:** 10.1371/journal.pone.0191535

**Published:** 2018-01-16

**Authors:** The PLOS ONE Staff

Dr. Soo Jung Park should be indicated as a Corresponding Author. Her contact information is: Taorgi@hanmail.net.

In the Funding section, the role of the funders is incorrect. The correct funding information is as follows: This work was supported by the National Research Foundation of Korea (NRF) funded by the Ministry of Education (2015R1D1A1A01058744, 2016R1A2B4009614) and the Korea Institute of Oriental Medicine (K17060). The National Research Foundation of Korea (NRF), funded by the Ministry of Education, designed hypothesis and supervise experiments, collected and analyzed the data. The Korea Institute of Oriental Medicine decided to publish, wrote the manuscript and analyzed the data.
