# Supplementary material for: Cordycepin promotes apoptosis in renal carcinoma cells by activating the MKK7-JNK signaling pathway through inhibition of c-FLIPL expression
Source: PLoS One. 2017 Oct 18;12(10):e0186489. doi: 10.1371/journal.pone.0186489 (PMC5646797; doi:10.1371/journal.pone.0186489)
Supplement: S1 Table — TK-10 cells were xenografted to the left and right thighs of nude mice and treated with cordycepin for 20 days at a mean tumor volume of 0.2 cm3. The volume of the tumor was determined on a 2-day interval using three-dimensional ultrasonography (Philips IU22 Ultrasound). The maximum volume for no treated with codycepin was 0.64 cm3 on the right. When treated with cordycepin, both side were significantly reduced to 0.001 cm3. There was no significant change in body weight when measured at 2-day intervals. (DOC) [file pone.0186489.s001.doc]

**S1 Table. Animal health monitoring with maximum size of tumor.**

|  | **No Treat** | | | **Cordycepin** | | |
| --- | --- | --- | --- | --- | --- | --- |
| **Day** | **Left**  **(cm^3^)** | **Right**  **(cm^3^)** | **Weight**  **(g)** | **Left**  **(cm^3^)** | **Right**  **(cm^3^)** | **Weight**  **(g)** |
| 0 | 0.222 | 0.299 | 21.1 | 0.230 | 0.226 | 21.2 |
| 2 | 0.253 | 0.329 | 21.4 | 0.156 | 0.188 | 21.5 |
| 4 | 0.281 | 0.361 | 21.6 | 0.135 | 0.156 | 21.8 |
| 6 | 0.311 | 0.398 | 21.6 | 0.129 | 0.123 | 21.7 |
| 8 | 0.340 | 0.433 | 21.3 | 0.117 | 0.084 | 21.7 |
| 10 | 0.371 | 0.465 | 21.3 | 0.099 | 0.055 | 21.9 |
| 12 | 0.401 | 0.496 | 21.1 | 0.051 | 0.037 | 22.1 |
| 14 | 0.428 | 0.533 | 20.8 | 0.028 | 0.017 | 22.6 |
| 16 | 0.457 | 0.564 | 20.9 | 0.009 | 0.008 | 22.9 |
| 18 | 0.489 | 0.599 | 20.6 | 0.002 | 0.004 | 22.9 |
| 20 | 0.517 | 0.644 | 20.7 | 0.001 | 0.001 | 23.1 |
